# Medical students' knowledge and attitudes toward history of medicine

**DOI:** 10.18502/jmehm.v13i6.4071

**Published:** 2020-08-25

**Authors:** Alireza Salehi, Hourieh Afsharipur, Hossein Molavi Vardanjani, Mina Vojoud, Leila Bazrafkan, Mohammad Hossein Sharifi

**Affiliations:** 1 Associate Professor, Research Center for Traditional Medicine and History of Medicine, Shiraz University of Medical Sciences, Shiraz, Iran.; 2 Researcher, Research Center for Traditional Medicine and History of Medicine, Shiraz University of Medical Sciences, Shiraz, Iran.; 3 Assistant Professor, MPH Department, Shiraz Medical School, Shiraz University of Medical Sciences, Shiraz.; 4 Researcher, Research Center for Traditional Medicine and History of Medicine, Shiraz University of Medical Sciences, Shiraz, Iran.; 5 Assistant Professor, Medical Education Department, Medical Education Development Center, Shiraz University of Medical Sciences, Shiraz, Iran.; 6 Assistant Professor, Research Center for Traditional Medicine and History of Medicine, Shiraz University of Medical Sciences, Shiraz, Iran.

**Keywords:** Medical students, Medical education, History of medicine, Iran.

## Abstract

Attention to the history of medicine (HM) has been increasing enormously among the scientific community. History of Culture and Civilization of Iran and Islam (HCCII) is taught in medical schools as a required course. However, data on medical students' level of knowledge and attitude about HM is limited.

This is a cross-sectional survey conducted between 2016 and 2017. A multi-stage random cluster sampling was done in which 230 medical students were asked to fill a standardized self-administered questionnaire. Univariate statistical tests and ordinary multivariable linear regression were applied.

Medical students' knowledge level was 50.8%, which is considered fair and weak. Interestingly, the knowledge score of those who attended only in HCCII course did not differ significantly from those who did not attend this course (*P* = 0.163). The results showed that knowledge scores were considerably greater in those who participated in related volunteer workshops than those who did not (*P* = 0.0001). The mean score of attitude toward HM was significantly higher in female subjects than male subjects (*P *= 0.028). Moreover, data indicated that attendance at the HCCII course and workshops was not associated with improvement in attitude.

According to the outcomes, the authors recommend revising the content, teaching method and structure of the HCCII course curriculum.

## Introduction

Knowledge of the History of Medicine (HM) is valuable for scientists and experts to open up new aspects of medicine (1). The history of science shows how societies have changed and evolved from ancient times to the present in medical science, revealing its great values in the community. It is possible that knowledge of HM can also prevent repetition of past mistakes in science because progress is made through error detection (2). In addition, by applying our knowledge of the past history of medicine, we can improve the future.

Exploration of and attention to HM to motivate the scientific community has been taken into consideration for several decades (3). In Europe and the United States, there are specialized medical journals on HM dating back several decades ago; for example, "Journal of the History of Medicine and Allied Sciences " has been published since 1946 (4). Iran is rich in experiences, opinions and medical beliefs due to its ancient civilization as well as its long history in medicine. The history of medicine in Iran has been an important part of the history of both the country and the world as a result of the contribution of Iranian people to the growth and completion of medical sciences for centuries (5, 6).

Although some medical schools do not pay enough attention to teaching HM and physicians might never have the time to study it, this field has grown rapidly in recent years (7). A lot of researchers and physicians in many countries have become progressively interested in HM (3,8). Also, teaching HM has long been a scientific subject in the field of medical education. For instance, the university of Birmingham medical school started to teach this subject in the medical curriculum during the academic year 1996 - 1997 (9), and Historical Discussion was presented as a seminar for fourth-year medical students at the University of Arkansas in the 20^th^ century (10). The medical education program was also expanded at Stanford Medical School in 2000 (11). Moreover, History of Medicine is available in the first year of the medical curriculum at Minnesota Medical School (12).

HM is not a part of medical school curriculum in Iran, but is included in the History of Culture and Civilization of Iran and Islam (the HCCII course). It seems that the main goal of HCCII is to raise the level of knowledge and change the attitude of students and the medical community. Based on previous studies, the HCCII course has pros and cons ranging from agree to completely disagree among faculty members as well as medical students (13).

According to the obtained data, the levels of knowledge and attitude about HM have not been studied in medical students so far. The aim of this study is to determine medical students’ level of knowledge and attitude about history of medicine. Moreover, there is an attempt to know whether the information received by medical students during the HCCII course affects their knowledge and attitude about history of medicine. Researchers have not investigated knowledge and attitudes about HM in much detail, and since the topic is very essential due to the growing importance of medical history, this study was conducted for the first time in Iran.

## Method

A cross-sectional study design was used among medical students of Shiraz University of Medical Sciences (SUMS). The participants were the medical students studying in all academic years from October 2016 to October 2017. Any medical student in SUMS during the mentioned period of time was eligible for inclusion in this study.

The sample size was determined by considering the size of the society (1200 people). Two hundred and sixty-three (263) medical students were selected using the Cochran statistical formula (a confidence interval of 95% and power of 80%). Based on their academic years, subjects were divided into two educational categories: basic sciences level (academic year 1 - 4) and clinical courses level (academic year 5 - 7). The sample size was selected proportional to the volume from each category. After the convincing sampling method was performed, two expert researchers explained the purpose of the research to the subjects.

It should be added that all medical students must take the HCCII course between the 2^nd^ and 3^rd^ academic years. However, it is voluntary to participate in workshops such as Persian Medicine (PM) and History of Medicine (HM).

***Data Collection and Questionnaire***

Since there was no standard questionnaire in this field, data collection was done through a researcher-made questionnaire. Ten questions were prepared about knowledge and ten about attitude; the questionnaire was developed during 3 meetings with three 3 expert faculty members. Finally, content validity of the questionnaire was confirmed by four expert faculty members of SUMS. The reliability of the questionnaire was also measured by a test-retest method on 20 medical students and repeated within an interval of two weeks, and Cronbach's alpha was measured at 0.79.

The questionnaire was classified into three components. The first part included demographic information, academic level, and participation in the HIICC course, PM workshop and HM workshop. The second part of the questionnaire consisted of 10 questions regarding knowledge and 10 about attitude. The knowledge section consisted of seven Likert scale questions (4 = very high, 3 = above average, 2 = average, 1 = below average, 0 = very low) and 3 Yes/No questions (correct answer = 1 and false answer = 0). The third part, i.e. the section regarding attitude, consisted of ten Likert scale questions (4 = strongly agree, 3 = agree, 2 = undecided, 1 = disagree, 0 = strongly disagree). Notably, the 2 attitude questions were negatively designed to determine the accuracy of the responses in completing the questionnaire.

In order to take account of ethical considerations, the questionnaires were anonymous and were identified by code. Confidentiality was maintained and the students participated in this research willingly. Ethical approval was received from Shiraz University of Medical Sciences (98-01-64-20586).

***Data Analysis***

Mean and standard deviation (SD) of knowledge and attitude scores were presented. Independent t-test, ANOVA, and simple linear regression tests were used to analyze the data using SPSS software version 19. A significance level of 0.05 was applied. According to quartile participant knowledge scores, the knowledge level was divided into four cutoff points including knowledge scores of 14 - 30 (very good), 9 - 13 (good), 6 - 8 (fair), and 0 - 5 (weak). According to quartile participant attitude scores, the attitude level was divided into four cutoff points including attitude scores of 29 - 39 (very good), 26 - 28 (good), 21 - 25 (fair), and 0 - 20 (weak).

## Results

Table 1 shows the demographic characteristics of the medical students who had attended the HCCII course, PM workshop, and HM workshop. Data indicated that 63.9% of the students were in basic sciences level. One hundred and thirty-three participants were male (~ 50%), and the average ages of the participants who were in basic and clinical level were 20.45 ± 0.20 and 24.6 ± 0.23 years, respectively.

**Table 1:** Demographic characteristics of the medical students based on academic level

Academic Levels Number (%)	Gender	Number (%)	Mean Age (SD)	Attendance at the HCCII Course, PM and HM Workshops (Yes - No)		Number (%)
**Basic Sciences ** **Level** **168 (63.9)**	Male	88 (52.4)	19.63 ± 0.173	HCCII course	Yes	15 (17)
					No	73 (83)
				PM workshop	Yes	7 (8)
					No	81 (92)
				HM workshop	Yes	8 (9)
					No	80 (91)
	Female	80 (47.6)	20.65 ± 0.216	HCCII course	Yes	32 (40)
					No	48 (60)
				PM workshop	Yes	2 (2.5)
					No	78 (97.5)
				HM workshop	Yes	4 (5)
					No	76 (95)
**Clinical ** **Level** **95 (36.1)**	Male	45 (47.4)	24.98 ± 0.154	HCCII course	Yes	45 (100)
					No	0
				PM workshop	Yes	7 (15.6)
					No	38 (84.4)
				HM workshop	Yes	16 (35.6)
					No	29 (64.4)
	Female	50 (52.6)	24.54 ± 0.320	HCCII course	Yes	48 (96)
					No	2 (4)
				PM workshop	Yes	4 (8)
					No	46 (92)
				HM workshop	Yes	11 (22)
					No	39 (78)

*HCCII: History of Culture and Civilization of Iran and Islam;* *PM: Persian Medicine; HM: History of Medicine.*

Among the participants, 50.3% (n = 140) reported that they had passed at least one unite of HCCI during their education.

**Figure 1:**
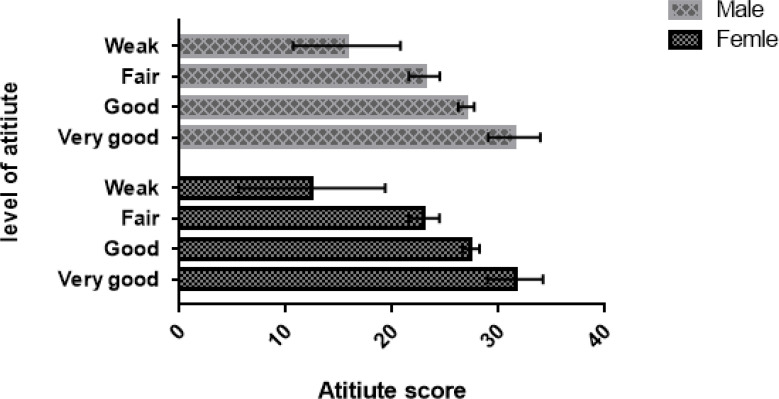
Level of atitiute in medical students

**Figure 2:**
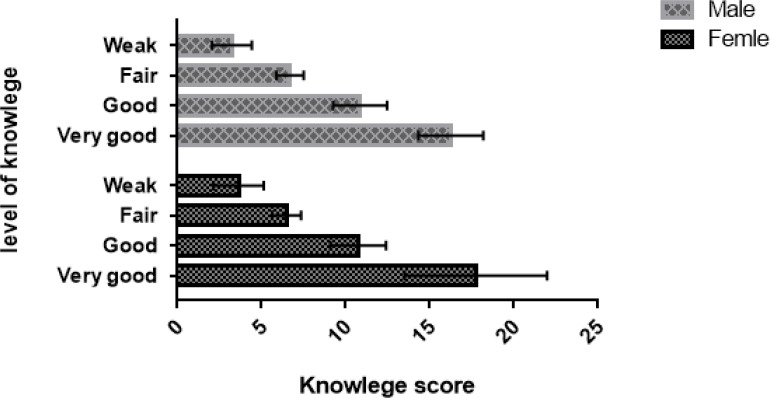
Level of Knowledge in medical students

The level of knowledge in both male and female students was 19.4% (very good), 29.8% (good), 19.8% (fair), and 31.0% (weak). But the level of attitude was 22.3% (very good), 21.5% (good), 26.0 (fair), and 30.2% (weak). Figures 1 and 2 show the details of knowledge and attitude levels in male and female students, separately.

Table 2 reveals the association of student’s knowledge and attitude score with independent variables. Although the results did not show significant differences between male and female participants’ knowledge scores (*P* = 0.205), there was a significant difference between their attitude scores (*P *= 0.028), in that it was higher in female subjects. The results also showed that there was a significant difference between the knowledge scores of those who had participated in the optional volunteer workshops of PM and HM and those who had not (*P *= 0.0001 and *P *= 0.0007, respectively). However, the knowledge scores of those who had participated in optional volunteer workshops were higher, but the knowledge scores of those who had participated only in the HCCII course did not differ significantly from those who had not (*P* = 0.163). Interestingly, the students' attitude scores decreased after attending the HCCII course, even though not significantly (*P* = 0.109). The students’ attitude scores decreased significantly after participation in the PM workshop (*P<0.001*), and their attitude scores also decreased after participating in the HM workshop, but this was not a significant decrease (*P* = 0.144).

**Table 2:** The mean ± SD student’s knowledge and attitude

Item	Subgroup	Number	Knowledge		Attitude	
			*Mean ± SD*	*P* *	*Mean ± SD*	*P* *
**Gender**	Male	133	9.22 ± 0.48	0.205	22.34 ± 0.76	0.028
	Female	130	8.39 ± 0.43		24.47 ± 0.57	
**HCCII Course**	Yes	140	9.23 ± 0.49	0.163	22.71± 0.69	0.109
	No	123	8.13 ± 0.40		24.28 ± 0.66	
**PM Workshop**	Yes	20	14.84 ±1.94	<0.0001	17.42 ± 2.52	0.001
	No	213	8.43 ± 0.31		23.80 ± 0.48	
**HM Workshop**	Yes	39	11.36 ± 1.03	0.0007	21.72 ± 1.54	0.144
	No	194	8.33 ± 0.32		23.70 ± 0.49	

* *Independent T-Test. PM: Persian Medicine; HM: History of Medicine; HCCII: History of Culture and Civilization of Iran and Islam.*

Tables 3 and 4 show the association of knowledge and attitude with independent variables in an adjusted regression model. In this model, students who attended the PM workshop were 7.9 times more likely to have “very good knowledge” than those who did not. The likelihood was significantly different from those who did not participate in this workshop (*P *= 0.014). Also, there was a 3.6 times greater chance for students attending the HM workshop to have “very good knowledge” than those who did not. The likelihood differed significantly from those who did not attend this workshop (*P* = 0.027). Students who were taking basic sciences were also 3 times more likely to have “very good knowledge” than those in clinical levels (*P* = 0.045). Nevertheless, data indicated that attendance at the HCCII course, PM workshop, and HM workshop was not associated with improving medical students’ attitudes (Table 4).

**Table 3:** Correlates of knowledge levels with independent variables in the final adjusted regression model

Independent Variables	Subgro up	Fair Knowledge		Good Knowledge		Very Good Knowledge	
		Adjusted OR 95% CI	*P*	Adjusted OR 95% CI	*P*	Adjusted OR 95% CI	*P*
**Gender**	Male	1.804 (0.840-3.876)	0.130	1.054 (0.541-2.053)	0.877	1.291 (0.562-2.966)	0.547
	Female	1		1		1	
**HCCII Course**	Yes	0.975 (0.345-2.757)	0.962	0.699 (0.264-1.848)	0.470	1.456 (0.494-4.297)	0.496
	No	1		1		1	
**PM Workshop**	Yes	3.623 (0.604-21.749)	0.159	1.828 (0.313-10.673)	0.503	7.938 (1.509-41.756)	0.014
	No	1		1		1	
**HM Workshop**	Yes	0.961 (0.245-3.766)	0.954	2.628 (0.952-7.254)	0.062	3.685 (1.162-11.690)	0.027
	No	1		1		1	
**Academic ** **Levels**	Basic sciences	2.264 (0.771-6.647)	0.137	0.948 (0.367-2.449)	0.912	3.026 (1.026-8.928)	0.045
	Clinical	1		1		1	

* Multinomial logistic regression; The reference category is weak; PM: Persian Medicine; HM: History of Medicine; HCCII: History of Culture and Civilization of Iran and Islam.

**Table 4:** Correlates of attitude levels with independent variables in the final adjusted regression model

Independent Variables	Subgroup	Fair Attitude		Good Attitude		Very Good Attitude	
		Adjusted OR 95% CI	*P*	Adjusted OR 95% CI	*P*	Adjusted OR 95% CI	*P*
**Gender**	Male	0.619 (0.300-1.276)	0.619	0.486 (0.225-1.050)	0.066	0.752 (0.349-1.622)	0.468
	Female	1		1		1	
**HCCII Course**	Yes	1.355 (0.499-3.675)	0.551	0.641 (0.209-1.961)	0.435	1.154 (0.402-3.311)	0.790
	No	1		1		1	
**PM Workshop**	Yes	0.696 (0.218-2.219)	0.696	0.152 (0.018-1.292)	0.084	0.566 (0.154-2.087)	0.393
	No	1		1		1	
**HM Workshop**	Yes	0.883 (0.319-2.443)	0.811	1.084 (0.346-3.396)	0.890	1.021 (0.343-3.034)	0.971
	No	1		1		1	
**Academic ** **Levels**	Basic sciences	1.033 (0.400-2.666)	0.946	0.694 (0.233-2.072)	0.513	1.239 (0.445-3.447)	0.681
	Clinical	1		1		1	

* Multinomial logistic regression; The reference category is weak; PM: Persian Medicine; HM: History of Medicine; HCCII: History of Culture and Civilization of Iran and Islam.

## Discussion

Medical students’ Information on knowledge and attitude toward history and medical history is limited. The findings showed that half of the students in HM did not have desirable knowledge and attitude, and the HCCII course, as required in the educational curriculum, had no effect on improving the attitude or knowledge of medical students.

Studies on the knowledge and attitude of medical students toward complementary medicine somewhat include HM because traditional medicine is a part of complementary medicine (14). We found numerous articles in the literature study that emphasized the value of teaching HM to medical students. Studies showed that most medical students were in favor of introducing complementary medicine and entering these subjects in the academic curriculum (15, 16). In Iran, application of complementary medicine to various diseases is increasing, and over the past decade, traditional Persian medicine has progressed as part of the complementary medical curriculum in universities (17). It is essential to evaluate the achievement of the goals of each course or seminar in order to modify the structure or content of the curriculum. However, the authors of the study did not investigate medical students' knowledge and attitude about HM in literature. 

There are various approaches to teaching HM at medical science universities (18). In England, medical students attend an HM course in the second academic year at Birmingham University and will present a paper on this topic the next year (9). Likewise, the Northwestern University Feinberg School of Medicine is offering a seminar on the history of medicine as part of its educational curriculum. In recent decades, the HCCII course has been added to the medical education curriculum of medical students in Iran and now it is a required course for all medical students. This course comprises two major sections. In the first section, it introduces the background of the population’s culture, Islamic civilization, development of science in Islamic civilization, etc. The second section involves the background of Muslim achievements by researchers who lived a few centuries ago. Considering the topics covered in the HCCII course, it was expected that the level of knowledge and attitude of students who passed this course would be improved compared to the rest of the students. However, there was no research to evaluate the effect of the HCCII course on medical students' knowledge and attitude in Iran.

Students' knowledge of HM may be an important target in enhancing the self-esteem of any community (9). Medical students' knowledge of HM can be a crucial point in increasing their self-esteem. The findings of this research showed that only 49.2% of the students had a favorable level of knowledge concerning HM (50.4% in men and 47.8% in women). In other words, more than half of the medical students did not have a favorable level of knowledge about HM. It is noteworthy that the level of knowledge among the students who attended the required HCCII course did not differ significantly from those who did not. These results indicated that the HCCII course is probably not able to enhance the students’ knowledge to a desirable level. This may be due to a lack of qualified educators, educational resources and educational facilities, limited lecture times, and the inefficient organization of education. It is therefore essential to review and reconfigure the structure, content or manners of the HCCII course. Of course, the level of knowledge was higher among students who participated in voluntary educational workshops such as HM or PM than students who did not. This indicates that workshops have been effective in enhancing students’ knowledge and should therefore be held more often. These findings are consistent with those of Darby’s study (19), a comparison between three groups of students involved in obligatory courses and three groups of students involved in elective courses. The results showed that the increase in the knowledge of students who participated in elective courses was more favorable.

Different variables such as psychological, family, community and even financial factors affect individuals’ attitudes. In education, three important factors that can change attitudes are the personality of the audience, the skill of the educators, and the characteristics of the message. This research showed that 47% of the female and 40.7% of the male participants had a favorable attitude toward HM. It is noteworthy that attitude toward HM was lower in students who attended both elective workshops and obligatory courses than those who did not, but the difference was not significant. This suggests that these courses cannot influence the attitudes of students. However, Hackler (10) argued that teaching HM to medical students improved thinking, enhanced critical and analytical approaches, and increased their social knowledge and consequent attitudes. Sokol (20) also argued that HM education could enhance the students' interest in research and curiosity (21). The reasons for the findings of the present study are not clear, but there are a number of potential explanations for the results. Firstly, this may be linked to the way that history of medicine is taught and conveyed. Historical subjects covered in this program are only taught in the form of lectures and not interactively. Obviously, dynamic and communicative methods of teaching could enhance attitude and motivate learning. Second, teachers at PM workshops probably lecture about past physicians’ methods of diagnosis or treatment that are not compatible with the modern approach, without sufficient and convincing explanations for students. Finally, evidence indicates that students’ attitude in different eras of science tend to be mainly context-dependent (22). Hence, there are different factors that affect students’ attitude toward history of medicine such as gender, teachers, curricula, culture, socio-ecnoimc status and so on (22, 23). 

Given the fact that the knowledge of students in this research did not change considerably after attending the required courses, it is suggested that voluntary workshops be held in this area. Moreover, fundamental modifications in the content and structure of the required courses are clearly needed. One of the limitations of this study was a lack of similar studies to compare the results.

## Conclusion

Teaching HM to medical students can be a great opportunity in every country to increase the student's knowledge of medical science history. HM training may even be effective in students' intention to do research. Since the present study showed that only about half of the students had a relatively good attitude toward HM, there is a need to review the construct and content of the educational curriculum to enhance the quality of the course. While the involvement of students in HM workshops is helpful in enhancing their knowledge, further studies are required to establish this.

## Notes:

**Citation to this article:**

Salehi A, Afsharipur H, Molavi Vardanjani H, Vojoud M, Bazrafkan L, Sharifi MH. Medical students' knowledge and attitudes toward history of medicine. J Med Ethics Hist Med. 2020; 13: 6.

## References

[1] Pearson D, Gove S, Lancaster J (2001). History of medicine. Health Information and Libraries Journal.

[2] Waller J (2008). Lessons from the history of medicine. J Invest Surg.

[3] Bynum WF, Porter R (1997). Companion Encyclopedia of the History of Medicine.

[4] Fridenberg P (1946). Journal of the History of Medicine and Allied Sciences. Archives of Ophthalmology.

[5] Nayernouri T, Azizi MH (2011). History of medicine in Iran the oldest known medical treatise in the Persian language. Middle East J Dig Dis.

[6] Zargaran A (2014). Ancient Persian medical views on the heart and blood in the Sassanid era (224–637 AD). Int J Cardiol.

[7] Paterson GR, Neilson JB, Roland CG (1982). History of medicine. Can Med Assoc J.

[8] Cox C (2013). Discursive essay: a better known territory? medical history and Ireland. Proceedings of the Royal Irish Academy Section C: Archaeology, Celtic Studies, History, Linguistics, Literature.

[9] Arnott R (2002). The university of Birmingham medical school and the history of medicine. Medical Humanities.

[10] Hackler C (2003). University of Arkansas college of medicine, division of medical humanities. Acad Med.

[11] Shafer A (2003). Stanford university school of medicine, arts and humanities medical scholars program. Acad Med.

[12] Anonymous Medical school university of Minnesota curriculum.

[13] Shedlock J, Sims RH, Kubilius RK (2012). Promoting and teaching the history of medicine in a medical school curriculum. J Med Libr Assoc.

[14] Rosenbaum CC (2007). The history of complementary and alternative medicine in the US. Ann Pharmacother.

[15] Ameade EPK, Amalba A, Helegbe GK, Mohammed BS (2015). Medical students' knowledge and attitude toward complementary and alternative medicine–a survey in Ghana. J Tradit Complement Med.

[16] Akan H, Izbirak G, Kaspar EÇ (2012). Knowledge and attitudes toward complementary and alternative medicine among medical students in Turkey. BMC Complement Altern Med.

[17] Dastgheib L, Farahangiz S, Adelpour Z, Salehi A (2016 May). The prevalence of complementary and alternative medicine use among dermatology outpatients in Shiraz, Iran. Iran J Med Sci.

[18] Armocida E, Aldini NN (2018). Teaching and learning the history of medicine in the university: some considerations after the students' final exams. Medicina Historica.

[19] Darby JA (2006). The effects of the elective or required status of courses on student evaluations. Journal of Vocational Education and Training.

[20] Sokol DK (2008). Perspective: should we amputate medical history?. Academic Medicine.

[21] Bârsu C (2017). History of Medicine between tradition and modernity. Clujul Medical.

[22] Osborne J, Simon S, Collins S (2003). Attitudes toward science: a review of the literature and its implications. International Journal of Science Education.

[23] Donohue SK, Richards LG (2009). Factors affecting student attitudes toward active learning activities in a graduate engineering statistics course. Proceedings of 39th IEEE Frontiers in Education Conference.

